# Cytomegalovirus as a cause of hypertensive anterior uveitis in immunocompetent patients

**DOI:** 10.1186/s12348-016-0100-5

**Published:** 2016-09-09

**Authors:** Jin A Choi, Kyu Seop Kim, Younhea Jung, Hae Young Lopilly Park, Chan Kee Park

**Affiliations:** 1 Department of Ophthalmology and Visual Science, St. Vincent’s Hospital, College of Medicine, The Catholic University of Korea, 93-6, Ji-dong, Paldal-gu, Suwon, Kyonggi-do 442-060 Republic of Korea; 2 Department of Ophthalmology and Visual Science, Seoul St. Mary’s Hospital, College of Medicine, The Catholic University of Korea, Banpo-daero 222, Seocho-gu, Seoul, 137-701 Republic of Korea

**Keywords:** Glaucoma, Uveitis, Cytomegalovirus, Endothelium, Cornea

## Abstract

**Background:**

The aims of this study are to investigate the clinical characteristics of patients with anterior hypertensive uveitis and to compare the characteristics between patients in cytomegalovirus (CMV)-positive and CMV-negative groups in their aqueous humor samples.

Immunocompetent patients (*n* = 42) with a history of chronic and/or recurrent hypertensive anterior uveitis underwent ophthalmic examination and serological tests. Among the 42 patients with hypertensive anterior uveitis, aqueous humor sampling was performed in 21, and they were analyzed for viral deoxyribonucleic acids using the polymerase chain reaction (PCR).

**Results:**

The average age of the 42 patients with hypertensive anterior uveitis was 57.6 years, and 29 (69.0 %) of the subjects were males. Of the patients, 22 (52.4 %) underwent glaucoma surgery, and the average corneal endothelial cell counts were 1908 cells/mm^2^. Among the 21 patients who underwent an aqueous sampling, 6 were positive for CMV-DNA, while 15 were negative. The frequency of glaucoma surgery was similar between groups (CMV positive vs. CMV negative, 66.0 vs. 66.0 %, *P* = 0.701). However, 66.7 % of the CMV-positive group underwent glaucoma tube shunt surgery, whereas 80 % of the CMV-negative group underwent trabeculectomy or received an ExPRESS glaucoma filtration device (Alcon, Fort Worth, TX) for glaucoma surgery (*P* = 0.095). The corneal endothelial cell counts were significantly lower in the CMV-positive group (CMV positive vs. CMV negative, 1245 ± 560 vs. 1981 ± 387 cells/mm^2^; *P* = 0.009).

**Conclusions:**

CMV was found to be an etiological factor in patients with hypertensive anterior uveitis in Korea. Special caution is needed for patients with CMV-induced hypertensive anterior uveitis, considering its adverse effect on the corneal endothelium.

**Electronic supplementary material:**

The online version of this article (doi:10.1186/s12348-016-0100-5) contains supplementary material, which is available to authorized users.

## Background

Herpes viruses are known to play a role in the idiopathic anterior uveitis associated with ocular hypertension [1–4]. There are three herpes viruses responsible for ocular inflammation; human cytomegalovirus (CMV), herpes simplex virus (HSV)-1, and varicella zoster virus (VZV). CMV has been recognized as a cause of morbidity and mortality, mostly in immunocompromised individuals [3, 4]. Recent reports have shown that CMV infection is an emerging cause of anterior uveitis associated with ocular hypertension in immunocompetent subjects [1, 2, 5, 6]. CMV is also an important cause of corneal endotheliitis, particularly in Asian populations [7–12]. However, the pathogenesis of CMV-induced anterior uveitis in immunocompetent patients and the systemic and ocular characteristics of the disease are not well understood.

In the present study, we investigated the clinical characteristics of patients with anterior hypertensive uveitis and compared the ocular and systemic characteristics between CMV-positive and CMV-negative patients in their aqueous humor. Finally, we investigated the factors associated with the corneal endothelial cell loss in the hypertensive anterior uveitis patients.

## Methods

This was a retrospective review of patients with anterior hypertensive uveitis who were investigated at Seoul St. Mary’s Hospital from March 2009 to June 2014. This study was performed according to the tenets of the Declaration of Helsinki, and the study protocol was approved by the institutional review/ethics boards of the Seoul St. Mary’s Hospital, the Catholic University of Korea (IRB number: KC14RISI0513). All of the patients included in this study met the following criteria: (1) anterior uveitis with keratic precipitates (KPs) and (2) increased intraocular pressure (IOP). Patients with the following were excluded: (1) presence of inflammation in vitreous or retina and (2) presence of corneal endothelial changes for a known cause other than anterior uveitis.

All of the participants underwent a comprehensive ophthalmic examination, including a detailed review of medical and ocular histories, best-corrected visual acuity measurement, slit-lamp biomicroscopy, Goldmann applanation tonometry, specular microscopy using a non-contact specular microscope (Konan Noncon Robo, Konan Medical, Inc., Hyogo, Japan), dilated stereoscopic examination of the optic nerve head and fundus, stereoscopic optic disc photography and red-free retinal nerve fiber layer (RNFL) photography (Nonmyd 7; Kowa Company Ltd., Nagoya, Japan), achromatic automated perimetry using the 24–2 Swedish Interactive Threshold Algorithm Standard program (Humphrey Visual Field (VF) Analyzer; Carl Zeiss Meditec, Inc., Dublin, CA, USA), and optical coherence tomography scans (Cirrus OCT, Carl Zeiss Meditec) to measure peripapillary RNFL thickness. Peripapillary RNFL thickness was determined three times at 256 points around a set diameter (3.4 mm) circle using the fast RNFL program. Only well-focused, well-centered images without eye movement and a signal strength ≥7 were used. A global average RNFL thickness provided by the software was used for the analysis. All of the patients underwent a laboratory work-up (including complete blood count, erythrocyte sedimentation rate analysis, white cell count and differential, blood chemistry) and serologic screening for IgM and IgG anti-CMV, HSV, and VZV.

Among the 42 patients with hypertensive anterior uveitis, aqueous sampling was performed in 21. Using a 30-gauge needle, 100 μL aqueous humor was aspirated under aseptic conditions and subjected to a polymerase chain reaction (PCR) assay for CMV, HSV1, and HSV2 DNA. DNA was extracted from the aqueous humor samples using a QIAamp DNA minikit (Qiagen, Valencia, CA, USA). Quantitative CMV-DNA PCR testing was performed using an AccuPower CMV Quantitative PCR Kit (Bioneer, Daejun, Republic of Korea). For HSV PCR, the HSV 1/2 PCR Kit (Bio-Core, Seoul, Republic of Korea) was used.

Glaucoma was defined as having glaucomatous disc appearance (thinning of neuroretinal rim, peripapillary hemorrhage, or localized pallor) associated with a typical reproducible VF defect evident on standard automated perimetry. A glaucomatous VF defect was defined as a glaucoma hemifield test result outside normal limits and the presence of at least three contiguous points in the pattern deviation plot with *P* values <5 %, with at least one point associated with a *P* value <1 % on two consecutive reliable VF examinations.

Patients were classified according to the duration of active intraocular inflammation. Patients with at least 3 months of active intraocular inflammation were considered to have chronic uveitis, and other patients to have recurrent episode of acute uveitis, which was normalized between attacks [2]. Glaucoma treatment was started in a step-wise manner; anti-glaucoma medication (beta-blocker, alpha-2 agonists, topical acetazolamide, and prostaglandin), and finally, glaucoma surgery. Glaucoma surgery was chosen among conventional trabeculectomy/ExPRESS glaucoma filtration device (Alcon Laboratories, Fort Worth, TX, USA) or Ahmed glaucoma valve implant (New World Medical, Inc., Rancho Cucamonga, CA, USA) surgery, considering preoperative IOP and severity of glaucomatous optic disc damage.

### Statistical analysis

For independent samples, the non-parametric Mann-Whitney *U* test and the *χ*^2^ test were used to compare between-group means and percentages. Multivariable analysis was performed using simple and multiple linear regressions for corneal endothelial cell counts according to the presence of CMV-DNA in the aqueous humor. First, we adjusted for age and gender (model 1). Then we adjusted for age, gender, lens status, and history of glaucoma surgery (model 2). A *P* value <0.05 was considered to statistically significant. All of the statistical analyses were performed using the SPSS software (ver. 14.0 for Windows; SPSS Inc.).

## Results

The clinical features of our patients are summarized in Table 1. The average age of the 42 patients with hypertensive anterior uveitis was 57.6 years, and 29 (69.0 %) of the subjects were males. In total, 22 (52.4 %) patients underwent glaucoma surgery, and the mean corneal endothelial cell counts were 1908 cells/mm^2^.

**Table 1:** Clinical parameters of 42 patients with hypertensive anterior uveitis

Gender (M/F)	29:13
Age (range), years	57.6 (25–88)
Spherical equivalent, D	−2.6 (−11.25–0.00)
Glaucoma operation,%	22 (52.4 %)
Corneal endothelial cell count, cells/mm^2^	1908 (625–3067)
Unilaterality	39 (92.9 %)
KPs at baseline examination	28 (67.8 %)
Anterior chamber reaction with 1+ or less	37 (88.1 %)
Severe peripheral anterior synechiae	0 (0.0 %)
Typical feature of P-S syndrome	14 (33.3 %)

Among the 21 patients who underwent aqueous sampling, six were positive for CMV-DNA, whereas 15 were negative (Table 2). The CMV-positive group was significantly younger and more myopic than the CMV-negative group (CMV positive vs. CMV negative: 47.5 ± 14.8 vs. 67.6 ± 11.8 years, *P* = 0.006; −3.6 ± 4.2 vs. 0.0 ± 1.6 D, *P* = 0.031). The frequency of glaucoma surgery was similar between the groups (CMV positive vs. CMV negative, 66.0 vs. 66.0 %, *P* = 0.701). However, 66.7 % of CMV-positive group had an Ahmed glaucoma valve implanted, whereas 80 % of the CMV-negative group underwent trabeculectomy or use of the ExPRESS glaucoma filtration device (*P* = 0.095). The corneal endothelial cell counts were significantly lower in the CMV-positive group (CMV positive vs. CMV negative: 1245 ± 560 vs. 1981 ± 387 cells/mm^2^; *P* = 0.009).

**Table 2:** Comparisons of clinical and immunologic characteristics in subjects with or without CMV in aqueous humor

Characteristics	CMV-positive subjects *n* = 6	CMV-negative subjects *n* = 15	*P* value
Demographic characteristics			
Male, *n *(%)	6 (100 %)	13 (86.7 %)	0.500
Age, years	47.5 ± 14.8	67.6 ± 11.8	0.006
Ocular characteristics			
Initial BCVA	0.65 ± 0.29	0.58 ± 0.29	0.569
Final BCVA	0.47 ± 0.46	0.52 ± 0.30	0.733
Spherical equivalent, D	-3.6 ± 4.2	0.0 ± 1.6	0.031
Axial length, mm	25.7 ± 1.5	24.4 ± 0.7	0.053
Glaucoma, *n *(%)	6 (100 %)	13 (86.7 %)	0.347
Average RNFL thickness, μm	66.2 ± 16.7	77.8 ± 19.1	0.132
Corneal endothelial cell count, mm^2^	1245 ± 560	1981 ± 387	0.009
Unilaterality, *n *(%)	83.3	80.0	0.684
KPs at baseline examination, *n *(%)	83.3	73.3	0.550
Presence of PAS, *n *(%)	4 (66.7 %)	5 (33.3 %)	0.331
Anterior chamber reaction with 1+ or less, *n *(%)	100.0	92.9	0.714
Lens status, phakic, *n *(%)	5 (83.3 %)	5 (33.3 %)	0.055
Baseline IOP, mmHg	28.83 ± 8.25	22.73 ± 7.86	0.132
Maximum IOP, mmHg	37.17 ± 8.03	39.53 ± 13.02	0.622
Final IOP, mmHg	13.3 ± 3.44	13.5 ± 5.05	0.950
Number of anti-glaucoma medication, *n*	2.67 ± 0.81	2.67 ± 0.81	0.970
Course of uveitis			0.544
Chronic, *n *(%)	4 (66.7 %)	13 (86.7 %)	
Recurrent, *n *(%)	2 (33.3 %)	2 (13.3 %)	
Glaucoma operation, *n *(%)	4 (66.7 %)	10 (66.7 %)	0.701
Choice of glaucoma operation			0.095
Ahmed valve, *n *(%)	3 (75.0 %)	2 (20.0 %)	
Trabeculectomy or ExPRESS^ⓡ^, *n *(%)	1 (25.0 %)	8 (80.0 %)	
HSV PCR positivity, *n *(%)	0 (0.0 %)	1 (6.2 %)	0.727
CMV RQ PCR, copies/mL	46,048.8 ± 91,334.5	Negative	n/a

*CMV* cytomegalovirus, *BCVA* best-corrected visual acuity, *RNFL* retinal nerve fiber layer, *KP* keratic precipitate, *PAS* peripheral anterior synechiae, *n/a* not applicable

Table 3 shows the laboratory characteristics of subjects with and without CMV-PCR(+) in the aqueous humor. In the CMV-positive subjects, the percentage of monocytes tended to be lower than in the CMV-negative subjects, with marginal significance (*P* = 0.055), whereas other parameters did not differ between the groups. The presence of CMV in the aqueous humor was significantly associated with corneal endothelial cell count after controlling for age and gender (*P* = 0.023), which was maintained after additional adjustment for lens status and history of glaucoma surgery (*P* < 0.001; Table 4). The clinical information of the 6 CMV+ patients was described in the Additional file 1, and two representative cases with antiviral treatment are discussed.

**Table 3:** Comparison of laboratory findings in subjects with or without CMV-PCR (+) in aqueous humor

Characteristics	CMV-positive subjects *n* = 6	CMV-negative subjects *n* = 15	*P* value
Complete blood count			
WBC count, cells/μL	7300 ± 900	6,700 ± 1900	0.590
Seg, %	54.4 ± 8.7	55.9 ± 7.7	0.677
Lymp, %	35.0 ± 6.6	31.9 ± 7.1	0.424
Mono, %	6.5 ± 0.7	7.8 ± 1.6	0.055
Eosinophil, %	3.5 ± 1.8	3.8 ± 3.2	0.970
Basophil, %	0.6 ± 0.3	0.5 ± 0.2	0.302
RBC count, 10^6^/μL	5.0 ± 0.5	4.4 ± 0.6	0.059
Hemoglobin g/dl	15.5 ± 1.4	13.9 ± 2.0	0.070
Platelet count, 10^3^/μL	247.5 ± 51.0	220.8 ± 58.8	0.329
ESR, mm/h	10.5 ± 7.6	31.8 ± 32.7	0.283
Blood chemistry			
FBS, mg/dL	103.7 ± 18.7	100.5 ± 44.1	0.519
AST, IU/L	23.3 ± 3.3	23.6 ± 10.5	0.367
ALT, IU/L	28.7 ± 10.5	27.2 ± 15.7	0.590
BUN, mg/dL	15.3 ± 4.7	17.8 ± 6.7	0.261
Creatinine, mg/dL	0.9 ± 0.1	1.0 ± 0.3	0.971
GFR, ml/min/1.73 m^2^	91.1 ± 8.9	79.2 ± 19.1	0.178
Na, mEq/L	142.3 ± 1.2	141.9 ± 3.2	0.858
K, mEq/L	4.4 ± 0.4	4.3 ± 0.5	0.914
Immunology			
HSV IgM positivity, *n* (%)	1 (16.7 %)	0 (0.0 %)	0.273
HSV IgG, titer, AU/mL	3.3 ± 1.4	6.5 ± 6.8	0.320
VZV IgM positivity, *n* (%)	1 (16.7 %)	0 (0.0 %)	0.273
VZV IgG titer, AU/mL	5.3 ± 3.1	5.0 ± 2.2	1.000
CMV IgM positivity, *n* (%)	0 (0.0 %)	0 (0.0 %)	n/a
CMV IgG titer, AU/mL	54.7 ± 15.3	55.7 ± 16.4	1.000

*HSV* herpes simplex virus, *VZV* varicella zoster virus, *CMV* cytomegalovirus, *ESR* erythrocyte sedimentation rate, *FBS* fasting blood sugar, *AST* aspartate aminotransferase, *ALT* alanine aminotransferase, *BUN* blood urea nitrogen, *GFR* glomerular filtration rate, *n/a* not applicable

**Table 4:** Association between the presence CMV DNA in aqueous and corneal endothelial cell count in patients with anterior hypertensive uveitis

	Model 1	Model 2	Model 3
	Unadjusted	Adjusted for age and gender	Adjusted for age, gender, lens status, and history of glaucoma surgery
	*B* (CI)	*B* (CI)	*B* (CI)
CMV positivity	672.4	734.4	1034.2
	(77.0–1267.9)	(116.4–1352.4)	(631.3–1437.1)
*P* value	0.029	0.023	<0.001

*B* beta coefficient, *CI* confidence interval

### Case reports

#### Patient 1

**Fig. 1:**
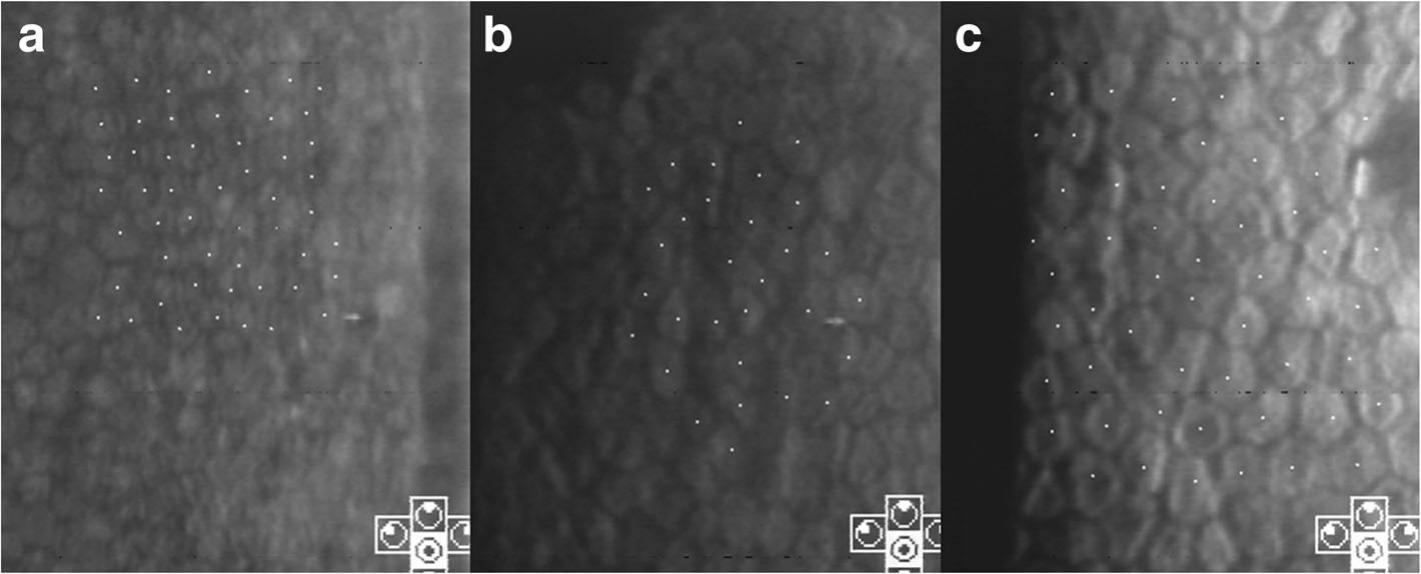
Case 1: serial follow-up of specular microscopy. At the initial visit, the corneal endothelial count in the left eye was 2141 cells/mm^2^ (**a**). After topical steroid and three antiglaucoma medications for 5 months, the corneal endothelial cell count decreased to 1245 cells/mm^2^ (**b**). A CMV-quantitative PCR analysis of aqueous humor samples revealed 229,000 copies/mL CMV-DNA. After valacyclovir treatment for 1 month, repeated anterior chamber tapping revealed 630 copies/mL CMV-DNA. However, the corneal endothelial cell count had decreased further, to 981 cells/mm^2^ (**c**)

A 68-year-old man had hypertensive anterior uveitis of his left eye with a recurrence rate of one to two times a month since 2010. On slit-lamp examination at Seoul St. Mary’s Hospital in October 2012, fine KPs in the lower part of the cornea and mild inflammatory reaction in the anterior chamber were seen. The corneal endothelial cell count of the left eye was 2141 cells/mm^2^, and the IOP was 22 mmHg with two topical antiglaucoma medications. He showed glaucomatous optic disc changes, with a mean deviation of −9.50 dB. Despite the use of topical steroids and three antiglaucoma medications for 5 months, his IOP was not controlled, and his corneal endothelial cell count in the left eye decreased to 1245 cells/mm^2^. A CMV-quantitative PCR analysis of aqueous humor samples for the left eye revealed 229,000 copies/mL CMV-DNA, but no HSV-DNA. Thus, an oral course of valacyclovir (90 mg/day) was started. The ExPRESS glaucoma filtration device (P-200) was implanted under a scleral flap. Oral valacyclovir (900 mg/day) was continued for 10 days, and anterior chamber tapping revealed 66,600 copies/mL CMV-DNA. Oral valacyclovir treatment (450 mg/day) was continued for another 14 days, and repeated anterior chamber tapping revealed 630 copies/mL CMV-DNA. After glaucoma surgery, the anterior chamber had a rare to +1 grade inflammation, and IOP was well controlled in a range of 6–9 mmHg. However, 5 months later, his corneal endothelial cell count was found to have decreased further, to 981 cells/mm^2^ (Fig. 1).

#### Patient 2

**Fig. 2:**
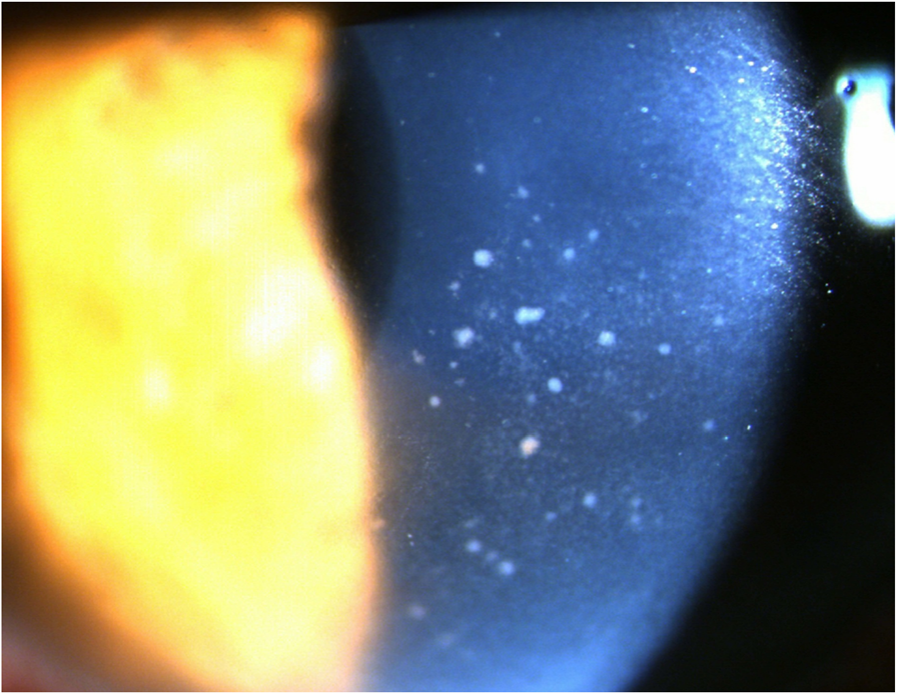
Case 2: slit lamp photograph showing diffuse mutton-fat keratic precipitates within the central area of the cornea. There was mild inflammation in the anterior chamber

A 64-year-old man had hypertensive anterior uveitis of his left eye with a recurrence rate of one to two times a month since 1991. He had been diagnosed with Posner-Schlossman syndrome, but chronic inflammation in the anterior chamber with uncontrolled IOP elevation was seen, and he was referred to Seoul St. Mary’s Hospital for glaucoma surgery. At the initial visit, mutton-fat keratic precipitates were noted in the central cornea with minimal anterior chamber reaction (Fig. 2). IOP was 21 mmHg with three antiglaucoma medications, and high pigmentation was noted in the anterior chamber angle. Severe glaucomatous optic disc was seen, with a mean deviation of −18.20 dB. The corneal endothelial cell count of the left eye was 1310 cells /mm^2^. A CMV-quantitative PCR analysis of an aqueous humor sample for the left eye revealed 44,609 copies/mL CMV-DNA, but no HSV-DNA. Oral valacyclovir treatment (900 mg/day) was started, and an Ahmed glaucoma valve was implanted. After 14 days of oral valacyclovir treatment, the aqueous humor showed 6980 copies/mL CMV-DNA. Oral valacyclovir was continued for 14 days, and further anterior chamber tapping showed 450 copies/mL CMV-DNA. After 1 month, the corneal endothelial cell count of the left eye was 1310 cells/mm^2^.

## Discussions

We reported the clinical characteristics of hypertensive anterior uveitis (Table 1), which occurred unilaterally (93 %), predominantly in middle-aged (57.6 ± 17.0 years) males (69.0 %; Table 1). Despite seemingly mild intraocular inflammation of the anterior chamber in the majority of patients (88.1 %) and the absence of peripheral anterior synechiae (0 %), glaucoma surgeries were required in over half of the patients (52.4 %). The male dominance in hypertensive anterior uveitis is consistent with previous results in non-Korean populations [4, 7]. Among the patients who underwent aqueous sampling, six (28.6 %) patients were positive for CMV-DNA. This prevalence is similar to the previous studies, reporting 22.8 % of anterior uveitis associated with raised IOP [1, 4].

Notably, the corneal endothelial cell count in the CMV-positive subjects was significantly lower than in the CMV-negative subjects (*P* = 0.009; Table 2). This association between the presence of CMV-DNA and the corneal endothelial cell count was more evident after adjusting for age, gender, lens status, and history of glaucoma surgery (*P* < 0.001; Table 3). Previous studies demonstrated that CMV is an important pathogen for chronic corneal endotheliitis, particularly in Asian populations [7–9, 13]. This study confirmed that CMV is a major cause of corneal endothelial cell loss in patients with hypertensive anterior uveitis.

Evidence of valacyclovir’s efficacy was seen in our study, using serial follow-up of CMV-DNA copy number and corneal endothelial cell counts in two case patients. After 1 month of therapy, CMV-DNA copy numbers decreased dramatically in both patients. Considering that patient 1 showed very high CMV-DNA copy numbers (44,609 copies/mL), it seems that one episode of acutely increased viral load is sufficient to cause extensive corneal endothelial cell loss. Consistent with our study, Kandori et al. [11] also reported a significant correlation between CMV viral load and corneal endothelial cell loss in CMV-associated uveitis.

We found that the proportion of monocytes tended to be lower in CMV-positive subjects, with marginal significance (*P* = 0.053; Table 2). Monocytes are the primary cell type of CMV persistence within the peripheral blood mononuclear cells [14]. CMV infection of monocytes induces trans-endothelial migration and monocyte to macrophage differentiation, which is productive for CMV replication [15]. Although the reason is unclear, CMV-induced differentiation of monocytes into macrophages may be associated with the lower proportion of monocytes in CMV-positive subjects in this study.

We found that the CMV-positive subjects were significantly younger (*P* = 0.006, Table 2). Generally, CMV seropositivity increases with age and serves as an immune risk phenotype, which is associated with survival in an older population [16, 17]. The current understanding of the mechanisms by which CMV reactivates in relatively young immunocompetent subjects is very limited. CMV is unlikely to cause clinically significant symptoms as long as it is maintained in balance by the host immune system [18]. However, recent literature suggests that CMV is associated with the pathogenesis of cardiovascular diseases, such as atherosclerosis, autoimmune disease, and even certain cancers [19, 20]. It is known that CMV can be reactivated when circulating monocytes with latent CMV are recruited to sites of inflammation [20]. For this reactivation, inflammatory cytokines, such as tumor necrosis factor and interferon, are known to play a role [21, 22]. In this regard, it is suggested that chronic inflammation in the anterior chamber may trigger CMV reactivation, which in turn, aggravates the inflammatory condition. In addition, CMV anterior uveitis is recalcitrant to topical steroid treatment [3, 4]. Recent reports show evidence that local immunosuppression by topical steroid use increases the risk of CMV infection in immunocompetent patients [23–26]. In addition, there is possibility that the frequent use of prostaglandin eyedrops have play an important role for worsening, including the uncontrolled glaucoma, considering previous reports regarding the complications from glaucoma eyedrops in uveitis [27–29].

## Conclusions

In conclusion, we found that CMV was a significant etiological factor in hypertensive anterior uveitis patients in Korea. In CMV-associated uveitis, extensive corneal endothelial cell damage may occur, even with effective anti-viral medication. CMV-associated hypertensive anterior uveitis patients were younger compared to CMV-negative uveitis patients. Special caution is needed for patients with CMV-positive hypertensive anterior uveitis, given its adverse effects on the corneal endothelium.

## Additional file

Additional file 1: Table S1. Demographic data and clinical manifestations of patients with the presence of cytomegalovirus in the aqueous. (DOCX 15 kb)
