# Supplementary material for: Cytomegalovirus as a cause of hypertensive anterior uveitis in immunocompetent patients
Source: J Ophthalmic Inflamm Infect. 2016 Sep 9;6(1):32. doi: 10.1186/s12348-016-0100-5 (PMC5017963; doi:10.1186/s12348-016-0100-5)
Supplement: Additional file 1: Table S1. — Demographic data and clinical manifestations of patients with the presence of cytomegalovirus in the aqueous. (DOCX 15 kb) [file 12348_2016_100_MOESM1_ESM.docx]

**Table S1** Demographic Data and Clinical Manifestations of Patients with the presence of Cytomegalovirus in the Aqueous

| Patient no. | age | gender | SE | Unilaterality | Initial IOP | Peak IOP | Lens status | Glaucoma surgery | Corneal endothelial cell count | Chronic/ recurrent | PAS |
| --- | --- | --- | --- | --- | --- | --- | --- | --- | --- | --- | --- |
| 1 | 39 | M | -3.0 | unilateral | 24 | 29 | Phakic | Ahmed | 1976 | Chronic | Yes |
| 2 | 27 | M | -11.25 | unilateral | 40 | 50 | Phakic | No | 2840 | recurrent | Yes |
| 3 | 62 | M | -2.1 | unilateral | 21 | 40 | pseudophakic | Ahmed | 1310 | Chronic | Yes |
| 4 | 67 | M | -0.3 | Bilateral | 22 | 32 | Phakic | Express | 1245 | chronic | No |
| 5 | 46 | M | 0.0 | unilateral | 38 | 39 | Phakic | No | 1295 | recurrent | Yes |
| 6 | 44 | M | -5.0 | unilateral | 28 | 42 | Phakic | Ahmed | 300 | Chronic | No |

SE: spherical equivalent; IOP: intraocular pressure; PAS: peripheral anterior synechiae
